# The Frailty Reduction via Implementation of Exercise, Nutrition, and Deprescribing (FRIEND) Trial: Study Protocol and Recruitment Results

**DOI:** 10.3390/mps7020026

**Published:** 2024-03-22

**Authors:** Michael Inskip, Carolina Almendrales Rangel, Chidiamara Maria Njoku, Fiona Barnett, Isabel Shih, Leonie O’Neill, Maria A. Fiatarone Singh, Trinidad Valenzuela

**Affiliations:** 1 College of Healthcare Sciences, James Cook University, Townsville, QLD 4814, Australia; 2 School of Health Sciences, Faculty of Medicine and Health, The University of Sydney, Sydney, NSW 2050, Australia; 3 The Good Shepherd Home, Townsville, QLD 4814, Australia; 4 Sydney Medical School, Faculty of Medicine and Health, The University of Sydney, Sydney, NSW 2050, Australia; 5 The Hinda and Arthur Marcus Institute for Aging Research, Hebrew SeniorLife, Boston, MA 02131, USA; 6 Exercise and Rehabilitation Sciences Laboratory, School of Physical Therapy, Faculty of Rehabilitation Sciences, Universidad Andres Bello, Santiago 7550196, Chile

**Keywords:** frailty, gerontology, aged care, exercise, nutrition, medication optimisation

## Abstract

Introduction: Virtually all adults in aged care facilities are frail, a condition which contributes to falls, cognitive decline, hospitalisation, and mortality. Polypharmacy, malnutrition, sedentariness, and sarcopenia are risk factors amenable to intervention. The Asia–Pacific Frailty Management Guidelines recommend anabolic exercise and the optimisation of medications and nutrition. However, no study has evaluated this best practice intervention triad in aged care. Methods: The Frailty Reduction via the Implementation of Exercise, Nutrition, and Deprescribing (FRIEND) Trial (ANZCTR No.ACTRN12622000926730p) is a staged 6-month translational trial evaluating resident outcomes, staff/caregiver knowledge, and institutional implementation in a Townsville aged care facility. Residents received high-intensity resistance exercise and balance training and medication and nutrition optimisation co-implemented by investigators (exercise physiologist, geriatrician, pharmacist, and nutritionist) and facility staff. Staff and caregivers completed comprehensive education modules and training. We report the trial protocol and recruitment results. Results: 29 residents (21 female, age: 88.6 ± 6.3 years) were recruited. At baseline, the residents were frail (frailty scale nursing home (FRAIL-NH); 6.3 ± 2.4/14), cognitively impaired (Montreal Cognitive Assessment; 13.8 ± 6.8/30), functionally impaired (Short Physical Performance Battery; 4.9 ± 3.1/12, 6 min walk distance; 222.2 ± 104.4 m), and were prescribed numerous medications (15.5 ± 5.9). Two residents died and one withdrew before the intervention’s commencement. Thirty family members and 19 staff (carers, allied health assistants, nurse managers, registered nurses, lifestyle–leisure officers, kitchen/hospitality staff, and senior leadership) were recruited to receive frailty education modules. Conclusions: The FRIEND trial is currently being implemented with results expected in mid-2024. This is the first trial to evaluate the implementation of the best practice frailty guidelines including anabolic exercise and medication/nutritional optimisation in residential aged care.

## 1. Introduction

Frailty is the most significant challenge to “ageing well” in Australia and affects virtually all older adults living within residential aged care facilities* [1]. Frailty reduces independence and quality of life and increases the risk of disability, falls, hospitalisation, and premature mortality with care costs doubling for every incremental increase in frailty [2,3]. Importantly, frailty is not an inevitable part of ageing. Instead, sarcopenia, undernutrition, decreased physical activity, chronic disease, and polypharmacy all contribute to frailty and are modifiable with appropriate treatment [2,4]. These treatments, backed by decades of clinical research and guidelines including the Asia–Pacific Clinical Guidelines for Frailty, include anabolic exercise, dietary fortification, and deprescription of inappropriate medications [2,5,6]. The formula is simple, yet this holistic approach to treatment has yet to become standard practice in residential aged care.

While nutritional or deprescribing initiatives in residential aged care facilities (RACFs) have been introduced in some, many barriers exist to sustainable implementation [7,8,9,10,11,12,13,14,15], and robust anabolic (resistance) exercise is virtually absent in this setting, particularly for those with both cognitive impairment and frailty [16]. Moderate-to-high-intensity progressive resistance training (PRT) has been shown to be superior to low-intensity resistance exercise for virtually every outcome targeted [17,18]. However, there is a substantial gap between this evidence and clinical practice due to misplaced fear of robust strength training and a lack of access to appropriate equipment and training protocols. Furthermore, implementing these interventions in isolation without fidelity to the guidelines represents a sub-optimal quality of care [19] which results in a sub-optimal quality of life [20,21,22,23,24] for the most vulnerable older adults. Thus, aged care interventions must not only be holistic to effectively treat frailty but also be implemented sustainably for long-term benefits in RACFs by equipping the sector with the knowledge and resources required.

The Frailty Reduction via the Implementation of Exercise, Nutrition and Deprescribing (FRIEND) Trial is the first study to fully implement these three components into one program in an RACF through co-implementation with facility staff. Furthermore, to increase sustainability in the long-term, the FRIEND trial will provide comprehensive education for staff, residents, and caregivers about the rationale and provision of evidence-based frailty assessment and treatment to improve outcomes for the most vulnerable in aged care.

### 1.1. Aim

The aim of the FRIEND trial was to establish integrated processes and pathways within a residential aged care facility that enable the early identification and effective reduction in frailty in residents with mild cognitive impairment (MCI) or dementia.

### 1.2. Research Objectives

#### 1.2.1. Primary Objectives

Effectiveness: To evaluate the impact of the implementation as assessed by measures of clinical outcomes and measures of experience of care.

Implementation: To examine the implementation outcomes including acceptability, adoption, knowledge transfer and fidelity, influences on uptake, integration, routinisation (structural-, organisational-, provider-, patient-, and innovation-level factors), and cost.

#### 1.2.2. Secondary Objective

The secondary objective was to establish mechanisms for the sustained implementation of the FRIEND trial within the residential aged care facility, as well as wide dissemination and implementation into other aged care facilities.

## 2. Methods

### 2.1. Study Design

The FRIEND trial (ANZCTR Registration No.: ACTRN12622000926730p [25]) was a staged 6-month, single-group pilot implementation research trial employing a mixed-methods research design to evaluate the process and impact of the implementation of best practice frailty management guidelines [2] for the FRIEND trial in the Good Shepard Home (TGSH) residential aged care facility (RACF), in Townsville, Australia. The trial received ethics approval from the University of Sydney Human Research Ethics Committee 25 July 2022 (HREC No: 2022/343) and reciprocal approval by James Cook University HREC (No C41, 23 September 2022) and TGSH board. The FRIEND trial was funded by the Dementia Centre for Research Collaboration (DCRC) ‘Implementing Research Evidence into Practice Grant’ and the James Cook University Philanthropic Chiropractic Research Fund.

### 2.2. Study Population

Participants for FRIEND consisted of aged care residents from three units within TGSH, their caregivers from the community, and staff with diverse roles from the facility including nurses, an exercise physiologist (AEP), care staff, allied health assistants, lifestyle–leisure officers, kitchen staff, and executive leadership including the Director of Care (DOC) and Chief Executive Officer (CEO).

### 2.3. Eligibility Criteria

#### 2.3.1. Aged Care Residents

Inclusion criteria: Men and women, age 60 or above, presence of cognitive impairment or dementia (Montreal Cognitive Assessment (MoCA) score < 26/30), presence of frailty (frailty status FRAIL-NH scale score > 1/14), able to understand written and spoken English. Exclusion criteria: Have permanent medical contraindications to the planned exercise due to unstable or terminal disease, unable to speak or understand instructions provided in English language, bed-bound, planning to relocate to a different area within the facility, or to a different facility within 7 months of trial enrolment.

#### 2.3.2. Family Member/Informal Caregivers of Participating Residents

Inclusion criteria: Men and women, age 18 or above, able to read and converse in English, able to visit their loved one at TGSH at least monthly throughout the duration of the trial, willing to support their loved one during their participation in the trial by (a) accompanying him/her during the study initial screening visit or connecting via video link or call; (b) attending an educational program (online) relating to the management of frailty and completing a pre- and post-knowledge questionnaire; and (c) attending a monthly 30 min case conference at TGSH or receiving care updates on clinical decisions.

Exclusion criteria: Unable to speak and understand English language at a conversational level.

#### 2.3.3. Staff Members

Inclusion criteria: Men and women, employed by TGSH in a role related to the provision of care for the participating residents, fluent English speaker.

Exclusion criteria: Unable to speak and understand instructions provided in English language.

### 2.4. Recruitment

Aged care residents and their caregivers were provided with information about the FRIEND trial in the form of leaflets and presentations, and facility staff (DOC) approached families to discuss interest in participating. Consent was gained by the resident and caregiver to be contacted by FRIEND investigators to perform baseline screening. Similarly, the staff at the facility who had a direct (i.e., clinical nurse manager) or indirect (i.e., kitchen staff) involvement with the three units selected for resident recruitment were also approached by the DOC, and their information passed onto the study investigators with consent to initiate the screening. The recruitment of all participants occurred between December 2022–March 2023.

### 2.5. Sample Size

The primary study population of interest was residents of TGSH aged care facility who were frail and had mild cognitive impairment (MCI) or dementia. Additionally, we aimed to recruit one family member/informal caregiver of each participating resident and at least 10 staff members from TGSH involved in the care of the participating residents.

The sample size was based on the primary effectiveness outcome of this pilot implementation study, using the nursing home frailty scale (FRAIL-NH scale). We expected residents to naturally become frailer over the course of a year without intervention. A longitudinal study of frail nursing home residents (n = 91) has reported an annual worsening (increased score) of 0.63 points without intervention [26]. In contrast, we hypothesised that our intervention involving progressive resistance and balance training, nutrition optimisation, and the deprescription of potentially inappropriate/unnecessary medications would improve the FRAIL-NH components of fatigue, ambulatory function, transfer function, and weight loss in nursing home residents due to expected improvements in strength, physical function, and walking reported in the literature with similar interventions [27,28,29]. We did not, however, expect our intervention to alter the FRAIL-NH items of incontinence, help with dressing, or the dietary modification/assistance required.

While we anticipated that an improvement in the four modifiable components may represent an increase of up to 4 points on the FRAIL-NH scale, we conservatively estimated that our holistic intervention would lead to an improvement (decreased score) of at least 2 points or one-half of this anticipated improvement in frail residents following 6 months of the staged implementation of all components compared to no change from baseline (again, a conservative estimate compared to Little 2021 [26] which suggested an increase (worsening) of at least +0.63 points would have occurred). Using the baseline standard deviation (SD) of 3.61 points for the FRAIL-NH scale reported by Little et al. [26] in nursing home residents (n = 91), we therefore estimated an improvement of a moderate effect size (Cohen’s d of 0.55). Using a two-tailed matched pairs t-test for our pre–post design with an alpha of 0.05 and beta of 0.2, we calculated that we would need to recruit 28 residents to our study.

### 2.6. Screening Procedure

Residents who provided informed consent to participate in the trial, and for whom consent was also attained from their caregiver (where required due to dementia), were scheduled to attend an initial screening visit to confirm their eligibility to participate in the trial. This visit took place at TGSH and lasted approximately 1 h. Information pertaining to demographics, medical history, medications, and restrictive practices as well as screening for the presence of frailty (score > 1/14 for FRAIL-NH) and MCI or dementia (MoCA ≤ 26/30) to determine eligibility was reviewed by the study geriatrician before enrolment. Caregivers and staff who met the eligibility requirements were enrolled once informed consent was gained.

### 2.7. Interventions

The FRIEND trial involved a comprehensive, multi-component intervention aimed at residents, caregivers, and facility staff. Primarily, the FRIEND intervention consisted of implementation of the best practice frailty intervention components for residents which included robust resistance and balance exercise and the optimisation of medications and nutrition. These interventions were co-facilitated throughout the intervention period by FRIEND investigators (inclusive of a geriatrician, AEPs, a nutritionist, and a pharmacist) and facility staff and guided by the development and implementation of comprehensive case conferences and reviews.

To support the successful implementation of these interventions for residents over the long term, the FRIEND intervention also involved comprehensive educational modules tailored primarily to facility staff and also for caregivers and residents in an adapted, briefer format. Details of the resident intervention and educational modules are below.

#### 2.7.1. Best Practice Frailty Interventions for Residents

##### Exercise Component of the FRIEND Intervention

The exercise component of the FRIEND trial aimed to improve muscle strength, balance, and mobility while also benefiting conditions such as cognitive impairment, depression, insomnia, hypertension, cardiovascular disease, and metabolic disorders. Thus, it was designed to also potentially reduce the number of medications or remove potentially harmful medications that residents may have been taking. Exercise can also help improve appetite, which may also contribute to better nutrition and a reduction in frailty.

Residents first underwent a comprehensive physical examination (~30–45 min in length) specifically developed to be conducted by an exercise professional (i.e., AEP) to evaluate readiness to undertake high-intensity progressive resistance training (PRT). The tool evaluated common musculoskeletal deficits and conditions (e.g., joint range limitations, posture, rotator cuff disease), neurological signs and symptoms (including parkinsonism, peripheral sensation, motor weakness), orthostatic blood pressure, and a thorough evaluation of skin integrity and peripheral circulation. The findings of this examination were then used to formulate considerations and modifications to exercise while also contributing to the case conference conducted for each resident.

Following the examination, residents attended up to two resistance and balance exercise training sessions per week on non-consecutive days. The sessions were delivered in a purpose-built gym at TGSH and supervised by an AEP, accompanied by exercise physiology students, and allied health assistants in a ratio of 1:1 to 1:3 depending on the level of cognition and function. The session durations were approximately 60–90 min, with balance exercises performed for the first 5–10 min of the session after pre-measures (blood pressure, heart rate) and PRT performed for the remaining part of the session.

Specific resistance exercises critical for mobility and function were prescribed for the upper and lower body using strength training machines [K400 Keiser pneumatic machines, Keiser Sports Health Equipment, Ltd., Fresno, CA, USA] as well as body weight, ankle cuffs, and free weights, depending on the capacity of each resident. Exercises were prescribed for 3 sets of 8 repetitions at 80% of the resident’s maximal capacity and included bilateral leg press, bilateral knee extension, seated row, and hip abduction. The intensity was increased over the first 2 weeks to approximately 80% of maximal capacity and then progressed throughout the 6-month intervention, guided by daily ratings of perceived exertion (15–18 on the Borg scale) [30], along with one-repetition maximum (1RM) strength testing every 6 sessions to maintain the intensity level at 80% of the current maximum capacity or above.

Balance training exercises aimed at reducing fall risk and improving functional capacity were also prescribed. Exercises included static (narrow, semi-tandem, tandem, one leg stance with/without perturbations) and dynamic balance exercises (heel-to-toe walking with/without perturbations and/or dual-task cognitive tasks) tailored to each resident’s balance ability. The intensity was increased continuously throughout the intervention to ensure the exercises remained challenging.

##### Nutritional Component of the FRIEND Intervention

The nutritional support component of the FRIEND trial aimed to enhance food and nutrient intake to improve nutritional status or mitigate malnutrition, thereby reducing frailty.

Targeted residents underwent a detailed assessment of their nutritional status, and they and the relevant staff received individualised nutritional recommendations. The nutritional recommendations included the removal of unnecessary or potentially harmful dietary restrictions that may increase the risk of unintentional weight loss or increase the risk of malnutrition. The research team reviewed the food/nutrient–drug interactions and exercise–diet interactions and advised the resident’s general practitioner (GP), when possible, on the potential deprescription of or dose reduction in any medication known to cause sedation, constipation, nausea, vomiting, anorexia, and/or diarrhoea. The supplementation of vitamin D was also included in the nutrition recommendations where indicated after an assessment by the resident’s treating GP. Protein intake should be between 1 to 1.5 g/kg body weight/day to maintain muscle in older adults [31]. Therefore, to increase protein intake and enhance skeletal muscle synthesis, a high protein snack was offered after the exercise sessions and at other times if needed. Nutritional recommendations were made to the staff and family of residents with malnutrition to increase nutrient density and improve nutritional status. To increase appetite, when possible, exercise was performed 1 h prior to a meal to stimulate appetite, and/or walking for at least 5–10 min before meals, when possible, was encouraged. An enhancement of the aesthetic aspects of food preparation and service and increased mealtime socialisation was used to promote dietary intake. This included, but was not limited to, the use of several strategies to improve appetite and meal enjoyment such as aromatherapy and enticing cooked meal smells (e.g., baked bread, popcorn) on each ward, volunteers deployed to socialise with residents while eating, and the establishment of a dedicated café zone adjacent to the clinical gym.

##### Medication Review Component of the FRIEND Intervention

The medication review component of the FRIEND trial aimed to identify, resolve, and prevent medication-related problems to reduce frailty and cognitive impairment. A medication review was carried out at baseline and in monthly intervals by the study’s pharmacist following the principles outlined within the quality use of medicines (QUM) and Residential Medication Management Review (RMMR) [32], which are endorsed by the Pharmaceutical Society of Australia.

The study’s pharmacist liaised with the facility staff to extract the medical history and current medication list of residents via the patient-centred database of The Good Shepherd Home (TGSH) for review by the study investigators. The resident’s GP was contacted to discuss all findings with an aim to reduce polypharmacy and drug interactions and side effects which may be contributing to frailty or cognitive impairment or other adverse health conditions. Established algorithms were used to identify excess dosages, drugs without sufficient indication, and potentially inappropriate medications (based on the BEERS list [33], STOPP protocols [34], anticholinergic burden, etc.).

The following steps were followed to guide the deprescribing protocol:

1. Ascertain indications of all drugs the resident was taking;
2. Consider the overall risk of drug-induced harm (drug–drug interaction, drug–exercise/nutrition interactions);
3. Assess each medication for its eligibility and whether it should be discontinued;
4. Prioritise drug discontinuation;
5. Monitor drug discontinuation regimens.

Medication use and management were optimised by ensuring that all current medications including vitamins and supplements had a valid current condition indication, and the overall risk of drug-related harm, cumulative risk from specific drug combinations (drug–drug interactions), drug–nutrient interactions, anticholinergic and sedative drug burden, drug side effects (nausea, vomiting, xerostomia/dry mouth, taste disturbances, altered bowel habits), and drugs contributing to falls, sarcopenia, osteopenia, gastrointestinal symptoms, and reduced exercise tolerance were considered. Undertreatment was also considered and managed while optimising formulations, and advice on adherence aids was given where necessary (combination formulations, etc.). Each participant was also evaluated for the use of any medications as part of restrictive practices in line with guidelines outlined by the Australian Aged Care Quality and Safety Commission [35].

A summary of recommendations based on the medication review was compiled into a holistic summary as well as the rationale and prescriptions for the exercise programming and the nutritional assessments and support suggested and sent to the residents’ GPs.

Lastly, an initial case conference was scheduled to discuss and implement the proposed modifications (as agreed by the resident’s GP) to the resident’s medication, exercise, and nutritional plans. The case conference was attended by the clinical nurse manager or nursing representative from TGSH, the study pharmacist, the study geriatrician, the resident’s GP, the resident, and the family member/informal caregiver (if available). Continuous monitoring for any adverse effects from discontinued or reduced dosages of medications, the exercise sessions, or any of the dietary alterations was undertaken by nursing staff as part of the usual care practices within TGSH and reported to the study physician (MFS) and the resident’s GP.

Monthly case conferences were planned thereafter with the clinical nurse manager to review resident progress and adherence to the treatment plans, evaluate how the different program components were being integrated, monitor changes to residents’ health status, and determine if any modifications needed to be made to the individualised FRIEND treatment plans. This monthly review also provided an opportunity to flag residents who required referral to their treating GP for further medical, pharmacologic, or nutritional evaluation.

#### 2.7.2. Educational Component of the FRIEND Intervention

##### Residents and Family Members/Informal Caregivers

Educational materials for participating residents and their families/informal caregivers were developed and delivered by the research team, in appropriate formats for each respective audience, to educate them on the role that exercise, nutrition, and deprescribing has on reducing frailty and improving the residents’ overall health. For the residents, a face-to-face session was delivered by FRIEND investigators accompanied by lay information flyers reviewed by consumers with cognitive impairment and caregiver advocates for appropriateness. Caregivers were given access to the FRIEND website and asked to complete educational modules consisting of up to four pre-recorded 30-min seminars on treatment for frailty targeted at a lay audience.

##### Participating Staff Members

An online educational program was delivered to participating staff members to educate them on the evidence behind the FRIEND trial and its application within the aged care context. The program was designed to be self-paced and take approximately 6 to 9 h to complete, and access to the educational program was provided via the FRIEND trial website and consisted of an introductory module on the overall roles that exercise, nutritional support, and deprescribing have on the management of frailty, followed by several in-depth modules on the different components of the FRIEND intervention and guides for implementation. Staff were provided with access to the modules relevant to their role at TGSH and provided with time during working hours to complete the required workload.

Participating residents, their family members/caregivers, and staff members were asked to complete a pre- and post-knowledge questionnaire before and immediately after completing the educational modules and then again at the conclusion of the FRIEND trial. The educational modules being developed as part of this translational trial will be reported upon in greater detail and available for dissemination following the completion of the trial in mid-2024.

#### 2.7.3. Rolling Implementation of Intervention Components

**Figure 1:**
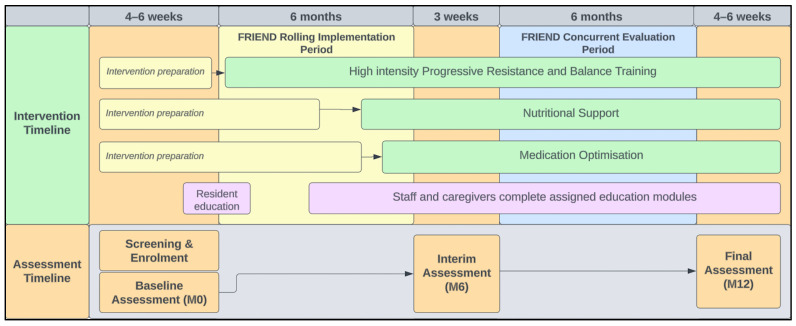
FRIEND intervention rolling implementation of components and assessment schedule.

The three components were staggered in their implementation to improve the allocation of resources and allow sufficient time to train staff as well as refine and strengthen processes. The exercise arm commenced in May 2023, being the most resource-intensive intervention, followed by the gradual implementation of medication and nutritional optimisation arms (October 2023) to allow sufficient time to prepare and discuss participant case conferences with staff and the residents’ GPs. The aim was to then evaluate a 6-month period of all three components running concurrently from October 2023 to May 2024. Figure 1 details the introduction of each intervention component and educational modules. The intervention baseline assessment (M0) denotes the commencement of the exercise component, while the interim assessment (M6) denotes the point in time when all three components and the education commenced, followed by the final assessment (M12) which takes place following six months of all three components being implemented concurrently (i.e., the FRIEND intervention).

### 2.8. Post-Trial Care

Post-trial care is detailed in Supplementary Materials.

### 2.9. Outcome Measures

#### 2.9.1. Study Participant Data

Qualitative and quantitative data were collected from three sources: the residents of TGSH, family members/informal caregivers of the participating residents, and staff members employed by TGSH in a role related to the provision of care for the participating residents.

The primary implementation outcome of interest relates to the implementation of the intervention which was measured in terms of acceptability, adoption, knowledge transfer and fidelity, and cost. The evaluation will also determine the preliminary efficacy of the FRIEND intervention when provided by the research staff in conjunction with the staff employed by TGSH. We will assess whether there were beneficial outcomes for the residents by comparing pre- and post-clinical outcome measures. Outcome measures and timepoints for the data collection are presented in Table 1.

#### 2.9.2. Facility-Wide Audit Data

Details of audit data are detailed in Supplementary Materials.

### 2.10. Analysis Plan and Dissemination Plan

Diverse quantitative and qualitative outcomes were collected during FRIEND. A detailed analysis plan for all outcomes and a dissemination plan is described in detail in Supplementary Materials.

## 3. Baseline Results

### 3.1. Recruitment

**Figure 2:**
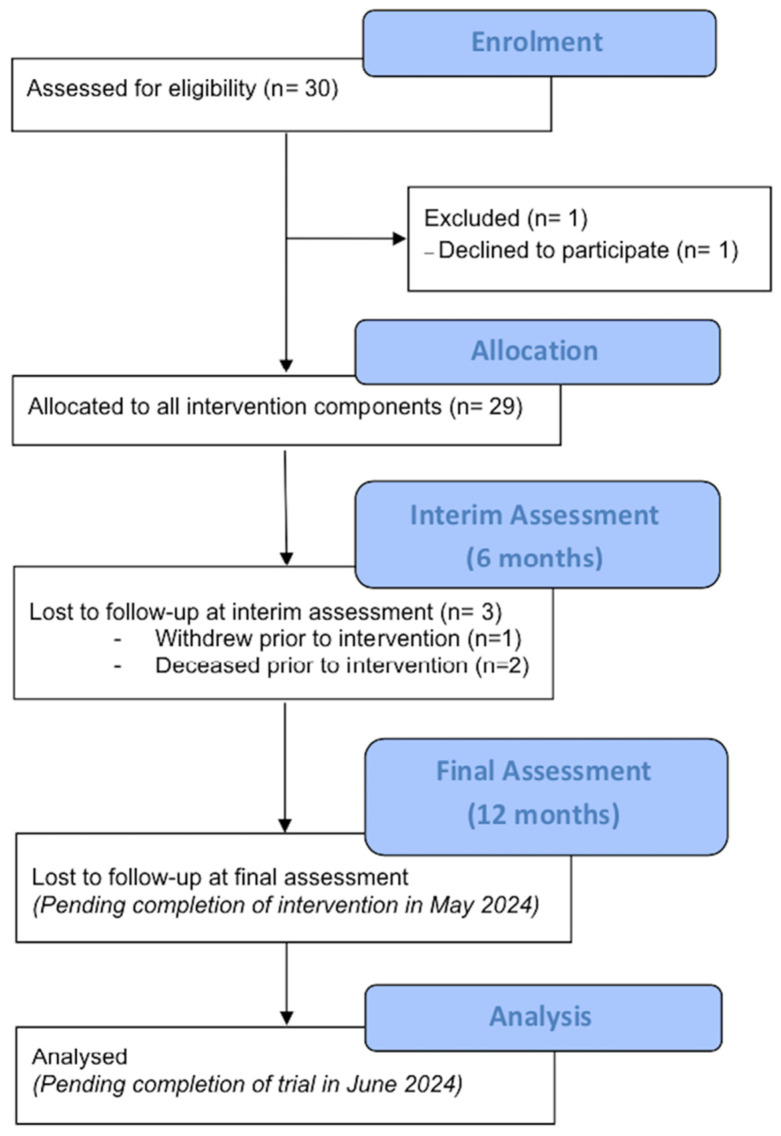
CONSORT flow diagram: recruitment flow of residents up until the interim assessment (6 months) timepoint.

The recruitment of the residents, caregivers, and staff began in December 2022 and concluded in March 2023. A total of 29 residents (detailed in a CONSORT flow diagram [44], Figure 2), 19 staff, and 30 caregivers were recruited during this period. All residents (but one) who were screened in the units were eligible and consented to join the trial. One resident withdrew from the trial prior to the completion of the baseline assessment, and a further two residents died (unrelated to the trial, n = 1 influenza-A and respiratory decline, n = 1 terminal dementia) prior to the intervention commencing.

### 3.2. Baseline Demographics

Table 2 details the demographic characteristics of residents, caregivers, and staff involved in the trial at baseline. The resident age ranged from 74 to 101 years of age, with the majority (70%) of the sample being female. All residents were of Caucasian descent (Australian, British, Italian, Greek, German). On average, residents in the FRIEND trial were moderately cognitively impaired and frail, with half of the sample meeting the definition of hyper-polypharmacy (≥15 medications prescribed) [45]. Additionally, all residents except for one were below the threshold score of 10/12 for the SPPB, indicating a higher risk of disability, adverse events, and mortality [46].

Most caregivers were female (73%) and were most commonly daughters, followed by sons. The staff had a range of experience in aged care environments and were predominantly female (90%) which is consistent with current industry estimates [47].

**Table 2:** Baseline resident characteristics.

Residents		
	Sample (*n*)	Mean (SD) or *n*
Age (years)	29	88.6 (6.3)
Sex (female/male), n		21/8
Body Mass Index (kgm^−2^)	27	26.2 (4.4)
Calf circumference (cm)	23	34.1 (3.0)
Time since admission to facility (months)		29 (4–182) *
Prescribed medications and polypharmacy Regular PRN Total prescribed Community definition (≥5), n Aged care definition (≥9), n Hyper-polypharmacy (≥15), n	29	10.7 (4.0) 4.8 (3.5) 15.5 (5.9) 24 21 15
Frailty FRAIL-NH (/14) Fried’s Frailty Phenotype (/5)	29 23	6.3 (2.4) 3.3 (1.1)
Cognition MoCA score (/30) Affect GDS-15 (/15) Significant depressive symptoms (>5/15, n)	29 26	13.8 (6.8) 4.3 (3.9) 10
Physical function and capacity SPPB (/12) 6MWT distance (m)	26 21	4.9 (3.1) 222.2 (104.4)
**Caregivers**		
	**Sample (*n*)**	***n***
Sex (female/male), n	30	22/8
Relationship to resident Spouse Daughter/daughter-in-law Son/son-in-law Sibling/sibling-in-law Close friend/acquaintance		0 18 8 2 2
**Staff**		
	**Sample (*n*)**	**Mean (SD) or *n***
Sex (female/male), n	19	17/2
Role Frontline Managerial Senior executive/leadership	19	12 5 2
Time working at facility (months)	17	72 (0–210) *
Time working in aged care (months)		72 (1–408) *

Footnote: * represented as median (range). Community definition of polypharmacy is commonly ≥ 5 medications [48], while in aged care in Australia, it is ≥9 medications [49], while ≥15 medications was used to denote what was considered hyper-polypharmacy [45]. All interpretations and references for the following measures provided in Table 1. FRAIL-NH—*Frail Nursing Home scale, MoCA Montreal Cognitive Assessment, GDS-15 Geriatric Depression Scale -15 Item, SPPB short physical performance battery, 6MWT six-minute walk test, PRN Latin for ‘pro re nata’, or medication prescribed as the need arises,* SD standard deviation.

## 4. Conclusions

The FRIEND trial is currently underway, and the concurrent implementation of the exercise, medication, and nutrition optimisation intervention components commenced in September 2023 and will continue until May 2024. Resident education has been completed, and caregiver and staff education are currently underway. As anticipated, the residents participating in the trial are frail, moderate–severely cognitively impaired, have low physical function and very high levels of polypharmacy, and are thus at a high risk of adverse outcomes such as falls, hospitalisation, and premature mortality [2] which underscores the importance of the FRIEND trial. While the trial is being conducted with a small sample, which may limit generalisability of the findings, it is a novel, important first step in pragmatically evaluating the translation of best practice frailty guidelines into aged care to treat frailty and its risk factors which will inform future, larger trials. Furthermore, if successful, the comprehensive suite of implementation resources developed during the trial will be disseminated freely following the conclusion of the trial to accelerate the implementation into the wider aged care sector internationally and be evaluated in future research. The results of the FRIEND intervention are anticipated in mid-2024, followed by the dissemination of educational materials.

## Figures and Tables

**Table 1:** FRIEND Trial outcome measures and timepoints.

Outcome	Question/Scope	Measure	Timepoint *
Implementation			
Acceptability	Experience of integrating the intervention	Semi-structured interviews with staff members, residents, and family members/informal caregivers.	M12
Adoption	Satisfaction with amount, content, and format of training provided	Number of staff, residents, and family members/informal caregivers who undertake the educational training modules.	M0–M12
		Questionnaire on experience of training and applicability of the skills learned—administered to staff, residents, and family members/informal caregivers.	M12
		Practice change via the Implementation Behaviour Survey administered to staff members.	M12
		Audit of intervention components delivered and resident adherence to the intervention components evaluated using self-designed intervention logs.	M0–M12
	Influences on uptake, integration, and routinisation (roles, relations, context, value systems, barriers/enablers)	Focus groups with managerial and senior representatives of TGSH regarding evaluation of system and process changes to embed the intervention within the organisation’s policies and procedures.	M0, M12
		Monthly implementation team meeting notes.	
		Semi-structured interviews with managerial staff and healthcare staff.	M12
Knowledge transfer and fidelity	Fidelity to key intervention features; adherence and outcome indictors	Fidelity to key intervention features evaluated via fidelity checks with research and staff members involved in the delivery of the intervention using self-designed fidelity checklists.	M0–M12
		Pre- and post-training knowledge acquisition quiz administered to staff, residents, and informal caregivers.	M0, M12
		Focus group discussions with consumer and consumer representatives, healthcare professionals, and stakeholders to provide feedback on the content of the FRIEND trial website which will be used for ongoing refinement.	M12
Cost	Cost of service implementation	Calculation of the cost involved with delivering the FRIEND intervention components (staff time, resources required).	M12
Preliminary effectiveness			
Frailty status	Changes in clinical outcome measures of residents	Frail Nursing Home scale (FRAIL-NH scale). Scored out of 14, with a higher score indicating greater level of frailty [36].	M0, M6, and M12
Frailty (Fried phenotype)		Derived from existing measures. Weakness (chair stand from SPPB), slowness (gait speed from SPPB), shrinkage (weight change from institutional data), fatigue (Q13 GDS-15), sedentariness (not meeting weekly exercise guidelines of 150/mins of at least moderate physical activity each week). Scored out of 5, with a score of 1–2 indicating pre-frailty and 3–5 indicating frailty [4].	
Nutritional status		Mini-nutritional Assessment (MNA)—including weight, height, mid-arm, and mid-calf circumferences. A score of 24–30/30 indicates normal nutrition, 17 to 23.5 indicates at risk of malnutrition, and <17 points indicates malnourished state [37].	M0, M6, and M12
Medication count and use of potentially inappropriate medications (PIMs)		Medication audit.	
Functional mobility and capacity		Short Physical Performance Battery (SPPB). Scored out of 12, with scores < 10 indicating greater risk of disability and adverse events [38]. Six-minute walk test (6MWT). Higher distance covered in a six-minute period indicates better function [39].	M0, M6, and M12
Maximal dynamic muscle strength		One-repetition maximum (1RM) strength on leg press and knee extension machines **	M0, M6, and M12
Quality of life		Quality of Life–Alzheimer’s Disease scale (QoL-AD scale). Scored out of 52, with a higher score indicating better quality of life [40].	M0, M6, and M12
Cognitive function		Montreal Cognitive Assessment (MoCA). Scored out of 30, with a higher score indicating better cognitive function and scores ≤ 26/30 indicating cognitive impairment [41].	M0, M6, and M12
Depressive symptoms		Geriatric Depression Scale—Short Form 15 Item (GDS-15). A higher score out of 15 indicates greater depressive symptoms, with a score > 5/15 indicating significant depressive symptoms [42].	M0, M6, and M12
Health status	Past medical history	Past medical diagnosis and symptoms (physical, mental, or emotional), past treatments, medications, past falls.	M0
	Ongoing health status	New medical diagnosis, new (or change) in symptoms, new (or change) in medications, falls, visits to healthcare practitioners. Gathered using a weekly health check questionnaire	Weekly
Clinical trial requirements			
Adverse events	All adverse events	All adverse events related and not related to the intervention. Gathered using weekly health status check and reporting of events throughout the study period.	M0–M12

Footnote: * rolling implementation of all three components of the intervention involves a staggered start with full implementation of all three components occurring between M6 and M12 for six months of combined intervention. M0: month zero (baseline), M6: month six (interim assessment), and M12: month twelve (final assessment). ** 1RM testing protocol followed the protocol described as gold standard for assessment of older adults [43].

## Data Availability

The raw data supporting the conclusions of this article will be made available by the authors on request.

## Supplementary Materials

The following supporting information can be downloaded at: https://www.mdpi.com/article/10.3390/mps7020026/s1.
