# Supplementary material for: The Frailty Reduction via Implementation of Exercise, Nutrition, and Deprescribing (FRIEND) Trial: Study Protocol and Recruitment Results
Source: Methods Protoc. 2024 Mar 22;7(2):26. doi: 10.3390/mps7020026 (PMC10961770; doi:10.3390/mps7020026)
Supplement: Supplementary file 1 [file mps-07-00026-s001.zip › mps-2887942-supplementary.pdf]

Supplement S1

S2.8. Post-trial care

The translational nature of the FRIEND project ensures that best-practice interventions are continued in a sustainable format for the facility. Following conclusion of the trial and the gradual withdrawal of FRIEND investigators, facility staff will continue to implement the interventions with resident participants and aim to expand the intervention to other residents of the facility beyond May 2024.

S2.9.2 Facility wide audit data

As part of the project and for the purpose of a) investigating the facility-wide prevalence of frailty and frailty related health outcomes, and b) contextualizing the sample of residents who take part in the FRIEND Study intervention, we collected cross-sectional data across all residents of The Good Shepherd Home (TGSH) via the Patient Centre Software as well as other data management software used at TGSH. Whenever possible data we acquired in a de-identified manner and included general demographic information (age, gender, ethnic background, years since admission); medical history (total number of medications, total number of diseases, prevalence of cognitive impairment and dementia, and prevalence of frailty); and nutritional status (height, weight, prevalence of special dietary requirements). In exceptional circumstances where data could not be provided in a de-identified manner, the research team de-identified the data as it is entered into the FRIEND Project database.

S2.10 Analysis Plan

S2.10.1 Quantitative analysis

All quantitative data was entered into SPSS. All outcomes were analysed with an intention-to-treat approach. Baseline sample characteristics were presented using descriptive statistics (mean and standard deviation). One way ANOVA was conducted to compare baseline study participant characteristics to the facility wide audit data. Intervention outcome measures were analysed via repeated measures Linear Mixed Models (LMM) or Generalised Linear Mixed Models (GLMM) as appropriate to the distribution of the data of baseline, 6-month and 12-month time points, adjusted for baseline values and all covariates selected a priori (age, sex, education) as well as any additional potential confounders associated with the dependent variable of interest identified. Mixed models were constructed to determine the TIME effect. We reported estimated marginal means (95% CIs), mean differences between timepoints and Hedges' bias corrected effect sizes (95% CIs) for all outcomes and accept significance at the 5% level. A p-value of <0.05 was used to reject the null hypothesis, and clinical meaningfulness of frailty results was compared to minimum clinically important differences (MCIDs) reported in the literature.

S2.10.2 Qualitative analysis

Focus groups and interviews conducted with staff members, residents and their informal caregivers were audio-recorded, transcribed verbatim and entered into QSR NVivo. Thematic analysis (developing codes) was used to identify patterns within the study groups. A combination of inductive and deductive coding was used. For residents and informal caregivers coding commenced with experience of the FRIEND program and perceptions of change but was open to unexpected findings that may have contributed to these. For staff members, frameworks focusing on implementation and organisational culture assisted to synthesise the data gathered in order to build a comprehensive assessment of the barriers and facilitators; and thus inform implementation.

S2.10.3 Implementation outcomes

Evaluation of implementation outcomes was guided by the framework of Normalisation Process Theory (NPT) which sought to understand how the intervention could be made workable and integrated into the organisational environment. NPT was utilised to identify and explain the factors that contribute to adoption of FRIEND. Four key components of NPT were used to inform implementation (including training provided), and evaluation (data collection and analysis): (i) Coherence (how do Nurses, Allied Health Professionals (AHPs) and General Practitioners (GPs) make sense of the intervention within the care they provide to residents; (ii) Cognitive Participation (will health care staff of the RACF have a commitment to implementing the intervention; (iii) Collective Action (what is the work required to ensure the intervention is implemented; and Reflective Monitoring (how did we appraise the benefits and costs of the intervention).

S2.11 Dissemination policy

The FRIEND website and materials developed for education and implementation of the interventions was refined using qualitative feedback received during the study period from participants. Following this process, funding will be sought to host and disseminate the comprehensive translational materials internationally and freely to other aged care facilities with appropriate copyright licensing.
